# The Cardioprotective Role of Nitrate-Rich Vegetables

**DOI:** 10.3390/foods13050691

**Published:** 2024-02-24

**Authors:** Beata Olas

**Affiliations:** Department of General Biochemistry, Faculty of Biology and Environmental Protection, University of Lodz, Pomorska 141/143, 90-236 Lodz, Poland; beata.olas@biol.uni.lodz.pl; Tel./Fax: +48-42-6354485

**Keywords:** beetroot, cardiovascular disease, nitrate, nitric oxide, vegetable

## Abstract

Nitric oxide (NO) is an inorganic radical produced by both the non-enzymatic nitrate (NO_3_^−^)—nitrite (NO_2_^−^)—NO pathway and enzymatic reactions catalyzed by nitric oxide synthase (NOS). Also, as nitrate and nitrite from dietary and other endogenous sources can be reduced back to nitric oxide in vivo, the endogenous NO level can be increased through the consumption of nitrate–rich vegetables. Ingestion of dietary NO_3_^−^ has beneficial effects which have been attributed to a subsequent increase in NO: a signaling molecule that may regulate various systems, including the cardiovascular system. A diet rich in NO_3_^−^ from green leafy and root vegetables has cardioprotective effects, with beetroot products being particularly good sources of NO_3_^−^. For example, various studies have demonstrated a significant increase in nitrite levels (regarded as markers of NO) in plasma after the intake of beetroot juice. The present review describes the current literature concerning the role of nitrate-rich vegetables (especially beetroot products) in the prophylaxis and treatment of cardiovascular diseases (CVDs). This review is based on studies identified in electronic databases, including PubMed, ScienceDirect, Web of Knowledge, Sci Finder, Web of Science, and SCOPUS.

## 1. Introduction

**Figure 1:**
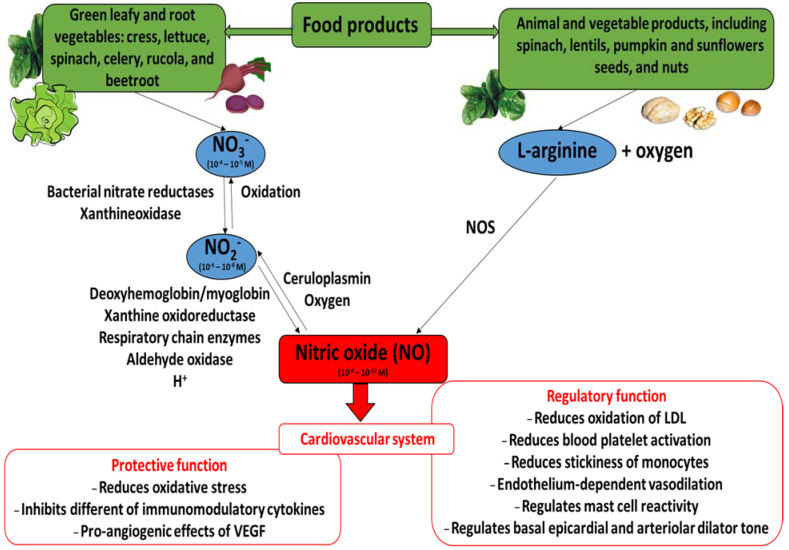
The nitrate (NO_3_^−^)/nitrite (NO_2_^−^)/nitric oxide (NO) pathways and beneficial action of nitric oxide in cardiovascular system. Concentrations market for NO_3_^−^, NO_2_^−^ and NO are those detected (NO_3_^−^ and NO_2_^−^) or estimated (NO) in blood ([7], modified).

Nitric oxide (NO) is an inorganic radical produced by both non-enzymatic and enzymatic reactions catalyzed by nitric oxide synthase (NOS; EC 1.14.13.39) (Figure 1). This enzyme exists as three isoforms: inducible nitric oxide synthase (iNOS), neuronal nitric oxide synthase (nNOS, constitutive form), and endothelial nitric oxide synthase (eNOS, constitutive form). Various cells may produce NO, including endothelial cells, neutrophils and macrophages. The conversion of L-arginine to L-cytruline by NOS (L-arginine—NOS pathway) is the primary source of nitric oxide, yielding about 70%. Also, NO can be synthesized through the nitrate (NO_3_^−^)—nitrite (NO_2_^−^)—NO pathway. When blood pH falls and oxygen-dependent NOS activity is limited, during ischemia for example, the formation of NO by the non-enzymatic reduction of nitrite/nitrate from dietary and endogenous sources becomes important. There is evidence that mammalian tissues have the capacity to reduce NO_3_^−^ to NO_2_^−^ via xanthine oxidoreductase or by the oral microbiome. However, humans have a greater proportional dependence on NO_3_^−^ reduction via the oral microbiome than by xanthine oxidoreductase compared with other mammals [1,2,3,4,5,6,7,8,9,10,11,12,13]. Interestingly, the reduction of nitrite by mitochondrial amidoxime reducing component (mARC) in the mitochondria may be an important signaling pathway for NADH-dependent hypoxic NO production [14,15].

The cardioprotective action associated with the consumption of fruits and vegetables have been attributed to their constituents, including minerals, fiber, vitamins and secondary metabolites. These cardioprotective properties may be also associated with the consumption of nitrate-rich food products [16,17,18,19,20,21,22,23,24]. Although vegetables represent the primary source of dietary nitrate (about 70–80% of intake), the European Food Safety Authority [25] report that fruits are also important sources, contributing 50% to 75% to the overall dietary intake. For example, a traditional Japanese diet is very high in nitrate from vegetables, and Japan has lower rates of coronary heart disease than the United States. It is also important to note that dietary patterns associated with blood pressure lowering, such as the Dietary Approaches to Stop Hypertension (DASH) diet is based around combinations of vegetables supplying about 160 mg of nitrate/day [24].

The present review describes the current literature concerning the role of nitrate-rich vegetables and their food products (especially beetroot products) in the prophylaxis and treatment of cardiovascular diseases (CVDs). The studies were identified in electronic databases, including PubMed, ScienceDirect, Web of Knowledge, Sci Finder, Web of Science, and SCOPUS. The last search was run on 12 February 2024, based on the following terms: “nitric oxide”; “nitrite”, “nitrate”, “beet”, “beetroot”, “cardiovascular system”, and “cardiovascular disease”.

## 2. Dietary Sources of Nitrate

**Figure 2:**
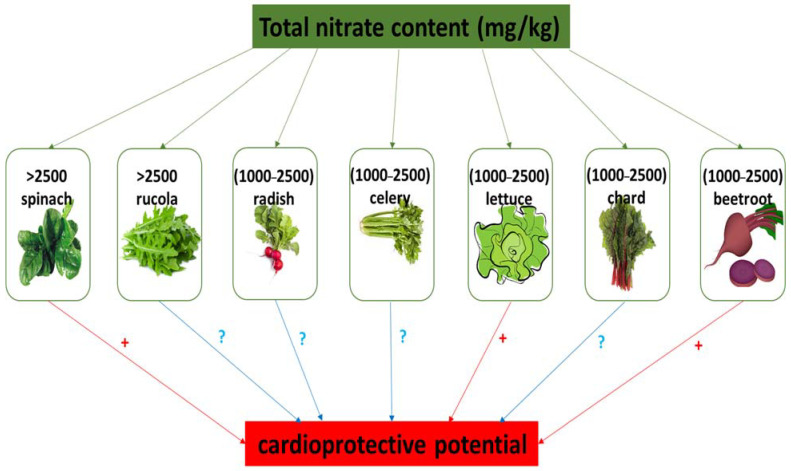
Nitrate content in selected vegetables and their cardioprotective potential. (+) cardioprotective potential; (?) no data about cardioprotective properties.

In humans, nitrate and nitrite reserves may be effectively increased through the consumption of green leafy vegetables and root vegetables, including beetroot, cress, lettuce, spinach, rucola, and celery, which may contain 1000 to 2000–2500 mg nitrate per kg^−1^ fresh weight [10,17,18,19]. In contrast, onions, peas, and potatoes contain low levels of NO_3_^−^. Also, leafy vegetables have higher levels of nitrate compared to tubers and seeds. Various dietary sources of nitrate and their classification based on nitrate content is presented in Table 1. In addition, Figure 2 shows the nitrate content (mg/kg) in selected vegetables and fruits as described by The European Food Safety Authority (EFSA) [25]. The level of NO_3_^−^ in vegetables is dependent on different factors, including soil type, the intensity of sunlight, nitrate content in water and fertilizers, cooking procedures, transport methods, and storage conditions [10,17,19,26,27]. The results of Ding et al. [28] indicate that pickled vegetable (for example pickled beets) have a lower nitrate content compared to fresh vegetables.

After consumption, exogenous NO_3_^−^ is absorbed by the gastrointestinal tract and enters the systemic circulation. Upon reaching the salivary glands, nitrate re-enters the oral cavity via protein transporters. About 25% is taken up by the salivary glands and concentrated in the saliva [29,30]. The results of Van Velzen et al. [31] report that nitrate from nitrate-rich vegetables has high bioavailability, and there are reports of close to 100% absorption following digestion [29,30].

NO_3_^−^ administration may influence the efficacy of NO_3_^−^, given that NO_3_^−^-rich beetroot juice contains other bioactive compounds such as ascorbic acid, phenolics and betalains [26,32,33].

Recently, Cocksedge et al. [34] reported that independently increasing or lowering oral temperature or increasing oral pH significantly increased mean salivary NO_2_^−^ after NO_3_^−^ supplementation in healthy adults. In this experiment, seven healthy men consumed 70 mL/day of beetroot juice (which has about 6.2 nM NO_3_^−^) during six separate laboratory visits.

**Table 1:** Dietary sources based on their nitrate content ([10,18,35], modified).

Nitrate Content	Dietary Sources	References
	Vegetables	
Very high (>2500 mg/kg fw)	Celery, cress, chervil, lettuce, beetroot, spinach, rucola	[18]
High (1000 to 2500 mg/kg fw)	Celeriac, Chinese cabbage, endive, fennel, leek, parsley	[18]
Medium (500 to 1000 mg/kg fw)	Cabbage, dill, turnip	[18]
Low (200 to <500 mg/kg fw)	Broccoli, carrot, cauliflower, cucumber, pumpkin, chicory	[18]
Very Low (<200 mg/kg fw)	Artichoke, asparagus, garlic, onion, green bean, mushroom, pepper, potato, sweet potato, tomato, watermelon, apple, banana, grape, pear, orange, strawberry	[18,35]
Very low (<20 mg/kg fw)	Meat	[10]
Very low (5 mg per 100 g)	Water	[10]

Fresh beetroot fresh juice is most commonly used for nitrate supplementation, but it has a lower concentration compared to other beetroot products (Table 2). Nevertheless, various studies have demonstrated a significant increase in nitrite levels (a marker of NO) in plasma after intake of beetroot juice [23,28,29,30,31,32,33,34,35,36,37,38,39].

The stability of NO_3_^−^ in vegetables and their food products is important when considering their functional activity. Corleto et al. [41] studied the stability of NO_3_^−^ in beetroot juice and arugula juice for 32 days at different temperatures (25, and 4 °C). They observed that NO_3_^−^ degradation starts within 24 h at 25 °C.

## 3. Regulatory Limits of Dietary Nitrate and Nitrite

The World Health Organization (WHO) and European Food Safety Authority have established the Acceptable Daily Intake of nitrate as 3.7 mg/kg of body weight, and nitrite as 0.06 mg/kg of body weight [42,43]. These limits translate into about 222 mg/day (NO_3_^−^) and 3.6 mg/day (NO_2_^−^) for a 60 kg person. In line with these recommendations, the consumption of 400 g of various vegetables and fruits per day, assuming median nitrate concentrations, would provide about 157 mg NO_3_^−^ per day [17,19].

It is important to note that NO_3_^−^/NO_2_^−^ can also be used as additives in foods. Nitrates (potassium nitrate—E252, sodium nitrate—E251), and nitrites (potassium nitrite—E250, sodium nitrite—E249) are authorized as food additives in the European Union under Commission Regulation (EU) No 1129/2011. They are used to stabilize processed cheese and meat. For example, the maximum concentration of nitrite is 150 mg/kg in cheese and in uncooked meat [44]. The maximum level for nitrate in vegetables (including spinach, lettuce and rocket) is also laid down in regulation (EC) No. 1258/2011 (set in the EU), expressed as mg nitrate/kg fresh weights [44].

Nitrates also act as anti-nutrient compounds [45,46], which have direct and indirect effects ranging from mild reactions to death. For example, the main anti-nutrients in lettuce include not only nitrates, but also tannins, phytates, and oxalates; these are described in a review by Shi et al. [45]. A number of papers have studied the conversion of NO_3_^−^ and NO_2_^−^ into nitrosamines, which have carcinogenetic potential [46,47]. Interestingly, the ecological risks connected with high nitrate consumption indicate that high nitrate content in vegetable is beneficial due to high content of natural antioxidants (polyphenols, betalain pigments, vitamins, and other) that prevent the formation of nitrosamines [48].

## 4. Nitric Oxide and Cardiovascular System

Nitric oxide acts as a gasomediator in various biological systems [49]. In the cardiovascular system, it induces vasorelaxation and promotes cardioprotection [3]. Moreover, different papers describe the fact that nitrate supplementation (including green leafy and root vegetables) may have an effect on cardiovascular health. They may help regulate blood pressure, limit a the progression of atherosclerosis, and improve myocardial contractility in healthy subjects and patients with cardiovascular diseases [8,10,23,36,37,39]. Figure 1 presents the beneficial action of nitric oxide (its regulatory and protective functions) in the cardiovascular system, together with the nitrate/nitrite/nitric oxide pathways.

Obtaining NO from natural food products is a better option for avoiding certain side effects than supplementation (for example with L-arginine and L-citruline), which may have mild to moderate side-effects, including gastrointestinal disturbances, heartburn, headache, and palpitations [50].

## 5. Role of Vegetable Nitrate in CVDs

Diabetes, obesity, hyperlipidemia and hypertension are considered risk factors for CVDs, including coronary heart, peripheral arterial disease, and cerebrovascular disease. In addition, CVDs become increasingly prevalent with age [51]; this has been attributed to vascular dysfunction, including the stiffening of the large elastic arteries (for example, carotid arteries and aorta). The development of endothelial dysfunction is the major clinical antecedent to atherosclerotic diseases such as occlusive stroke, coronary artery disease, and peripheral artery disease [52,53,54].

Various clinical studies and epidemiological evidence indicate an intake of diet enriched in vegetables and fruits has a significant effect on the prophylaxis and treatment of CVDs. Consuming a healthy diet may be also a good strategy for preserving vascular function with aging, and some papers suggest that sodium nitrite and nitrate supplementation may also be effective [18,55,56,57,58,59,60,61]. For example, Sindler et al. [55,56], Flennor et al. [57] and Woodward et al. [61] found that three-week supplementation of drinking water with sodium nitrite (80 mg/day) reduced systemic vascular resistance in C57BL/6 mice. In addition, many of the biologically active phytochemicals (including NO_3_^−^) present in green leafy and root vegetables may bestow cardioprotective benefits by various mechanisms.

In humans, consumption of nitrate (as a meal or supplement) induces increased circulating NO_3_^−^ and NO_2_^−^. Moreover, chronic consumption of whole food NO_3_^−^ sources elevates plasma NO_3_^−^ and NO_2_^−^ concentrations which are comparable to beetroot juice [62,63]. A review by Karwowska and Kononiuk [44] indicate that NO_3_^−^ intake has a number of beneficial effects on the cardiovascular system such as triglyceride reduction, blood pressure regulation, and stroke and atherosclerosis prevention. Moreover, dietary NO_3_^−^ may also play an important role in improving cardiovascular risk factors, with beneficial effects observed on a reduction in blood platelet activation, including platelet aggregation [10]. These properties were observed in both healthy subjects and patients with obesity and hypertension. The effects were particularly visible when dietary NO_3_^−^ intake (between 68 mg/day and 1395 mg/day) was in the form of beetroot juice and breads, arugula juice, and spinach leaves and juice administered between two hours and 42 days [10]. For example, Ashworth et al. [58] observed that the consumption of two portions of high-nitrate vegetables daily resulted in a reduction in blood pressure in normotensive women.

Various dietary components may interact with nitrate and/or nitrite, interfering with their antihypertensive action. For example, thiocyanate competes with NO_3_^−^ for absorption by the salivary glands. Cigarette smoke may also impair the metabolism of NO_3_^−^. On the other hand, the consumption of foods rich in phenolic compounds may enhance nitrite reduction in the stomach, which, in turn, may boost the effect of NO_3_^−^ on blood pressure [64].

## 6. Beetroot Products

Beetroot (*Beta vulgaris* L.) belongs to the *Chenopodiaceae* family. It is grown in various countries, and is consumed as part of the normal diet. In addition, it is used in manufacturing as a food coloring agent (known as E162) [39,65]. It is a source of various important bioactive compounds: dietary fiber, minerals (sodium, potassium, copper, iron, zinc, phosphorus, calcium, and magnesium), phenolic compounds (for example, phenolic acids, and flavonoids), ascorbic acid, carotenoids and betalains, including betanin [39,40,66,67]. Most importantly, it is also considered as a valuable source of nitrate. However, traditional beetroot formulations, including cooked vegetables and fresh juice must be offered in large amounts to reach pharmacological nitrate concentrations [40,66]. Despite this, consumption of concentrated beetroot juice significantly increases NO_3_^−^ and NO_2_^−^ with peak concentration occurring one to three hours post-consumption [62,63]. Beetroot juice contains a high concentration of nitrate (up to 11.4 g/L) as compared to drinking water (<45 mg/L in European countries) [68]. Webb et al. [2] noted a significant increase in NO_3_^−^ and NO_2_^−^ concentration in plasma: NO_3_^−^ up to 182 ± 55 µM after one to two hours (equivalent to 550%), and NO_2_^−^—up to 373 ± 211 µM after two to three hours (equivalent to 400%).

Recently, Brzezinska-Rojek et al. [69] reported that a serving of fresh beetroot provides significantly more nitrates and nitrites than most daily portions of beetroot-based dietary supplements.

Other studies have examined the effects of other beetroot products, including fermented juice, powder, bread, chips, crunchy slices, gel, and cereal bars, as supplements in healthy subjects or patients [40,66,70,71,72].

Beetroot and its products provide a variety of health advantages and may help prevent and manage various diseases, including CVDs. In addition, taking 8 g of dried beetroot for 20 days has an effect on hematological parameters. For example, these results showed a mild increase in hemoglobin readings, a decrease in the total iron binding capacity, decrease in transferrin and increase in ferritin [40,66,70,71,72].

Other potential positive effects of beetroot supplementation on cardiovascular health include reduced blood pressure, increased blood flow, improved endothelial function, a reduced renal resistance index and others [73]. Various systematic reviews about the effects of beetroot juice on blood pressure indicate that its consumption may have a beneficial role in the prevention and treatment of hypertension [36,73,74]. For example, a meta-regression by Siervo et al. [75] demonstrated an association between a daily dose of inorganic nitrate and changes in systolic blood pressure. Beetroot juice supplementation was also associated with a significant reduction in systolic blood pressure in adults. This study included 254 participants [75].

A systematic review of 11 studies by Ocampo et al. [36] also showed that beetroot juice supplementation is an effective strategy that may reduce blood pressure in populations of healthy and hypertensive patients, probably through the nitrate/nitrite/nitric oxide pathway and secondary metabolites found in beetroot. However, this effect depends on age, sex, baseline blood pressure, body composition, and body weight. Even so, beetroot juice supplementation seems to improve blood pressure control throughout adult life (45+). There is also a better response after this supplementation in the population with body mass index (BMI) > 25 and when there is a high baseline blood pressure. A study by Kim et al. on postmenopausal women [76] found blood pressure to be reduced after consumption of beetroot juice. Another meta-analysis showed a two-week supplementation with 500 mL/day of beetroot juice to have a particularly beneficial effect on blood pressure, and that the effect of this supplementation generated better results, compared to those with a duration of one week [60,77]. For example, reduced blood pressure was achieved with 250 mL intake of beetroot juice daily over four weeks.

On the other hand, other studies do not demonstrate any such reduction after nitrate-rich beetroot supplementation [78,79,80,81]. For example, Perez et al. [81] observed no significant changes in blood pressure in during handgrip exercise after a single shot of beetroot juice versus placebo, and neither did Craig et al. [78].

Oxidative stress is known to be an important factor for the development of CVDs, and plant antioxidants often have cardioprotective effects. Clifford et al. [39] report that beetroot supplementation may serve as a useful strategy for protecting cellular components from oxidative stress (in vitro and in vivo), and that beetroot juice demonstrates comparable, or higher, antioxidant capacity compared to carrot or tomato juice, and to pineapple or orange juice. Only pomegranate juice had a higher antioxidant capacity based on in ferric reducing antioxidant power (FRAP) assay. The study tested ten commercially available vegetable and fruit beverages in the UK [32,39,82].

A few papers have suggested that nitrate supplementation increases vasodilatation in human skin following heat stress, but that NOS-dependent vasodilatation was not affected by nitrate supplementation [83,84,85,86,87,88]. Other research groups observed that beetroot, as a natural NO donor, may preserve or restore endothelial function. For example, Webb et al. [2] demonstrated that beetroot supplementation (500 mL/day) preserves brachial artery endothelial function in healthy participants. Joris and Mensik [83] also observed that beetroot juice (140 mL/day) improves postprandial endothelial function in overweight and slightly obese men. A systematic review and meta-analysis also demonstrated that inorganic nitrate and beetroot supplementation, including beetroot juice, was associated with beneficial effects on endothelial function. In addition, these effects appear to be reduced in older subjects and in subjects with greater cardiometabolic risk [89]. On the other hand, some papers indicate that beetroot products have no influence on endothelial function; for example, Kenjale et al. [90] indicated that beetroot juice supplementation (500 mL/day) has no effect on endothelial function in peripheral arterial disease patients.

A pivotal mechanism in the pathogenesis of various CVDs, including stroke and acute coronary syndrome, is blood platelet activation. McKnight et al. [91] reported that oral nitrate inhibits platelet activation in healthy volunteers, and that the formation of S-nitrosothiols may be involved in the inhibition of platelet activation. Richardson et al. [92] also indicated that nitrate (0.5 mmol and 2 mmol) increases gastric S-nitrosothiol concentrations and inhibits blood platelet activation in healthy subjects. Other authors also found dietary nitrate from beetroot juice to have an inhibitory effect on platelet activation induced by ADP and collagen treatment [2,77].

Beetroot may be consumed not only in juice form, but also a whole food, gel form, and incorporated into bread [12,71,93]. Capper et al. [12] suggest that beetroot in its whole form may be a source of nitrate, but NO_3_^−^ concentrations vary depending on various factors, including the time of harvest. In addition, they examined whether beetroot food is a good form of dietary nitrate that can reduce blood pressure and improve blood flow in both young and older adults. In this experiment, 24 healthy, non-smoking participants, i.e., 12 young (age: 27 ± 4 years) and 12 older (age: 64 ± 5 years), consumed whole cooked beetroot in three portions: 100, 200, and 300 g, on four separate occasions over a four-week period. The main finding was that while incremental doses of dietary nitrate reduced systolic and diastolic blood pressure in young participants, significant decreases were only observed with the highest dose in the older group. Moreover, none of the interventions modified microvascular blood flow in either tested group; however, all interventions increased plasma nitrate and nitrite levels in both groups.

In addition, Hobbs et al. [71] noted that intake of a beetroot-enriched bread (100 g/day) can augment marked improvements in intravascular function in young healthy men.

**Figure 3:**
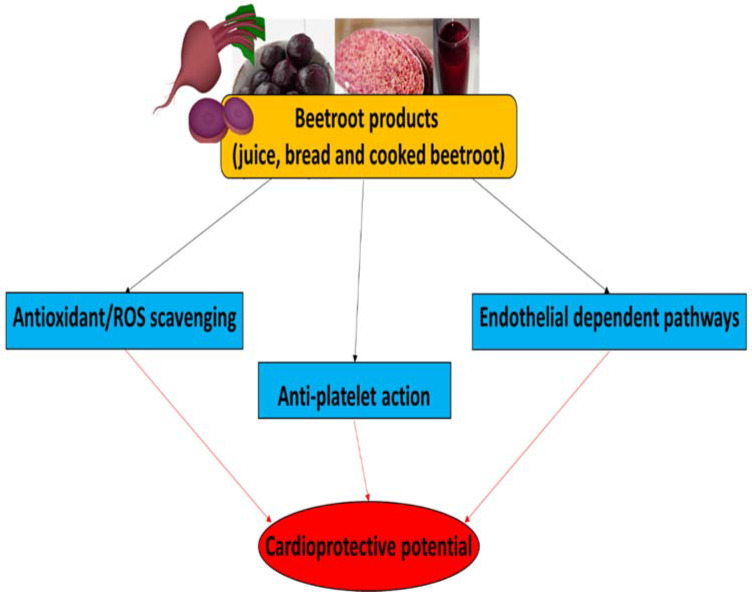
The therapeutic potential of beetroot products for CVDs and their main biological properties.

More information about the functional properties of beetroot, especially beetroot juice, in the management of cardio-metabolic diseases, including hypertension, insulin resistance, diabetes and kidney dysfunction, have been described in a review paper by Mirmiran et al. [94]. The therapeutic potential of beetroot products for CVDs, and their main biological properties, are summarized in Figure 3.

## 7. Spinach Products

Spinach (*Spinacia oleracea* L.) is a member of the family *Amaranthaceae* and is known for its dark green leaves. The family also includes chard and beets. About 82 unique varieties of spinach exist. Moreover, it is classified into three types according to the leaf texture: (I) savoy, (II) semi-savoy, and (III) smooth-leaf. Savoy and semi-savoy are used for cooking (for example soups, casseroles, and steaming), whereas smooth-leaf spinach is the preferred leaf type for salads, smoothies, and processing. In America, spinach is often consumed with other green leafy vegetables, such as cabbage (*Brassica oleracea* L. *var. capitate* L.), lettuce (*Lactuca sativa* L.), and broccoli (*Brassica oleracea* var. *italica* Plenck) [95].

Spinach is primarily composed of water (91.4%) and contains small amounts of lipids (0.4%, mainly mono- and polysaturated fatty acids), carbohydrates (3.6%), and proteins (2.9%). It also contains 2.2 g fiber, different vitamins and phenolic acids [95].

Various in vitro and in vivo studies on animals and humans indicate that spinach may protect against chronic diseases, including CVDs [95,96]. It is known to have various antioxidant, hypoglycemic, lipid-lowering, anti-obesity, antiproliferative, and anti-inflammatory properties [95,97]. For example, a recent study by Panda et al. [96] in which rats were treated with spinach mixture (400 and 800 mg/kg daily for 30 days), found spinach to demonstrate cardioprotective activity against isoproterenol-induced myocardial infarction in rats. In addition, Jovanovski et al. [98] studied the effect of 500 mL of spinach soup (845 mg nitrate/day for seven days) on arterial stiffness and blood pressure on 27 healthy participants. Their findings demonstrate that dietary NO_3_^−^ has promising potential to improve vascular health by decreasing arterial stiffness and blood pressure.

## 8. Lettuce and Other Nitrate-Rich Vegetable Products

Lettuce (*Lactuca sativa* L.), belonging to the *Asteracea* family, is a successful and diverse plant distributed worldwide. Lettuce is considered a particularly important leafy vegetable. It is rich in water (94–95% content) and low in calories. It is also a good source of minerals, vitamins, phenolic compounds and chlorophyll, but the phytochemical contents differ between the types of lettuce; for example, red lettuce has higher phenolic compound level than green [45,99,100,101,102].

Lettuce has a number of interesting impacts on CVD factors by different mechanisms owing to its fiber content and antioxidant availability [45,100,101,103]. For example, Nicolle et al. [103] noted a beneficial effect of lettuce consumption on lipid metabolism and on tissue oxidation in rats. Moreover, Abdalla et al. [104] observed that 20 mg/mL methanol extract from red lettuce has stronger radical scavenging activity than green lettuce based on free radical 2,2-diphenyl-1-picryl-hydrazyl-hydrate (DPPH) assay in vitro. Rolnik et al. [100] also indicated that preparations from green lettuce and red lettuce (*L. sativa var. crispa*) leaves have antioxidant properties in human plasma treated with a hydroxyl radical donor (in vitro model). The red lettuce leaf preparation also demonstrated anti-platelet potential in vitro, inhibiting blood platelet adhesion to collagen and fibrinogen. In addition, neither preparation was found to cause blood platelet lysis [101].

Kammoun et al. [105] report that *Ulva lactuce* ethanolic extract has hypolipidemic and cardioprotective actions in hypercholesterolemic mice. The tested extract alleviated cardiotoxicity, as shown by cell viability, heart oxidative stress, index of atherogenesis and plasma biochemical parameters. In addition, hypercholesterolemic mice supplemented with *U. lactuca* decreased expression of proinflammatory cytokines (tumor necrosis factor-α (TNF-α), intereleukins (IL-1β and IL-6)). Moreover, used extract had antioxidant activity in vitro.

However, none of the authors described the concentration of NO_3_^−^ in preparations and extracts from lettuce [100,101,103,105]. In addition, no studies have examined the relationships between cardioprotective action and NO_3_^−^ concentration among other NO_3_^−^ rich vegetables such as rucola, radish, celery and chard. For example, a review by Kooti and Daraei [106] only demonstrates that the antioxidant activity of celery is associated with various phenolic compounds, including apigenin, luteolin, and kaempferol.

## 9. Conclusions

Vegetable nitrate, especially that found in beetroot products (including juice, bread, and cooked beetroot) should be promoted as a key component of a healthy lifestyle aimed at controlling cardiovascular system function; however, there is also a need to study other factors related to supplementation with nitrate-rich vegetables, including secondary metabolites more deeply [107]. In addition, the cardioprotective mechanism by which NO_3_^−^-rich vegetable consumption remains unclear and poorly defined in the scientific literature.

The supplementation of beetroot products (especially, beetroot juice, bread and cooked beetroot) has been reported to reduce blood pressure, attenuate inflammation, decrease oxidative stress and inhibit blood platelet activation. However, there is a need for further clinical trials examining the cardioprotective potential and safety of nitrate-rich vegetables (not only beetroot, but also spinach, rucola, radish, celery, lettuce, and chard) and their products.

Like other vegetables, beetroot also contains a number of phenolic compounds which may have cardioprotective activity themselves as well as various synergistic effects.

## Figures and Tables

**Table 2:** Nitrate and nitrite contents of different beetroot products in 100 g of each products ([40], modified).

Beetroot Product	NO_3_^−^ (mmol)	NO_2_^−^ (mmol)
Cereal bar	14.0 ± 0.05	0.2 ± 0.01
Gel	6.3 ± 0.01	0.11 ± 0.02
Chips	6.9 ± 0.02	0.13 ± 0.02
Fresh juice	4.1 ± 0.01	0.1 ± 0.02

## Data Availability

No new data were created or analyzed in this study. Data sharing is not applicable to this article.

## Abbreviations

BMI—body mass index; CVDs—cardiovascular diseases; DPPH—2,2-diphenyl-1-picryl-hydrazyl-hydrate; EFSA—The European Food Safety Authority; FRAP—ferric-reducing antioxidant power; IL-interleukin; NOS—nitric oxide synthase; eNOS—endothelial nitric oxide synthase; iNOS—inducible nitric oxide synthase; nNOS—neuronal nitric oxide synthase; NO—nitric oxide; NO_3_^−^—nitrate; NO_2_^−^—nitrite; TNF-α—tumor necrosis factor-α; WHO—World Health Organization.
